# No impact of a high‐fat meal coupled with intermittent hypoxemia on acute kidney injury biomarkers in adults with and without obstructive sleep apnea

**DOI:** 10.14814/phy2.15804

**Published:** 2023-08-31

**Authors:** Nicholas Goulet, Emily J. Tetzlaff, Renée Morin, Jean‐François Mauger, Ruwan Amaratunga, Glen P. Kenny, Pascal Imbeault

**Affiliations:** ^1^ Behavioural and Metabolic Research Unit, School of Human Kinetics, Faculty of Health Sciences University of Ottawa Ottawa Ontario Canada; ^2^ Human and Environmental Physiology Research Unit, School of Human Kinetics, Faculty of Health Sciences University of Ottawa Ottawa Ontario Canada; ^3^ Institut du Savoir Montfort Montfort Hospital Ottawa Ontario Canada

**Keywords:** fatty acid‐binding proteins, hypoxemia, interleukin‐18, kidney injury molecule‐1, lipocalin‐2, oxygen saturation

## Abstract

Obstructive sleep apnea (OSA) is characterized by chronic intermittent hypoxemia, which is associated with progressive loss of kidney function, where postprandial fluctuations in renal physiology may further compromise oxygen supply and kidney function. Therefore, we measured biomarkers of acute kidney injury (AKI) following a high‐fat meal with and without intermittent hypoxemia. Eighteen healthy young men (mean age [SD]: 22.7 years [3.1]) and seven middle‐aged to older individuals with OSA (54.4 years [6.4]) consumed a high‐fat meal during normoxia or intermittent hypoxemia (~15 hypoxic cycles per hour, ~85% oxyhemoglobin saturation) for 6 h. We observed no changes in estimated glomerular filtration rate and plasma concentrations of creatinine, neutrophil gelatinase‐associated lipocalin (NGAL), and kidney injury molecule‐1 (KIM‐1) at any measured time points. In both groups, plasma concentrations of interleukin‐18 (IL‐18) increased after 6 h during normoxia only (*p* = 0.033, *η*_p_^2^ = 0.122), and plasma concentrations of liver‐type fatty acid‐binding protein (L‐FABP) transiently decreased after 3 h in both conditions (*p* = 0.008, *η*_p_^2^ = 0.152). These findings indicate that AKI biomarkers are not acutely elevated during the postprandial state with or without intermittent hypoxemia, suggesting that other mechanisms may play more important roles in the progression of kidney disease in OSA.

## 1 INTRODUCTION

Obstructive sleep apnea (OSA) is a chronic sleep disorder characterized by repeated episodes of complete or partial obstruction of the upper respiratory airway during sleep, leading to intermittent reductions in oxygen availability. The exposure to repeated cycles of deoxygenation‐reoxygenation leads to intermittent hypoxemia, which is considered the hallmark feature of OSA and is associated with many adverse health consequences (Turnbull, 2018). Accumulating evidence indicates that OSA is associated with kidney function loss and chronic kidney disease (CKD) (Hanly & Ahmed, 2014). For instance, polysomnographic studies report widespread sleep apnea among dialysis patients, with one study reporting rates of sleep apnea as high as 73% in patients with CKD (Kimmel et al., 1989). The relationship between OSA and kidney disease is further supported by improvements in cardiac hemodynamics and kidney health following continuous positive airway pressure treatment (Koga et al., 2013). Given the discrepancy between renal blood flow and the high oxygen demand of the medulla, the kidney is considered vulnerable to ischemic–reperfusion injury, leading to cell death, and reduced kidney function (Brezis & Epstein, 1993). Perturbations in oxygen delivery to the kidney may contribute to the progressive deterioration of kidney function in individuals with OSA, as clinical studies have independently associated the severity of intermittent hypoxemia with an increased risk of kidney function loss (Ahmed et al., 2011).

Besides intermittent hypoxemia, impairments in lipid metabolism may also play a role in explaining the link between OSA and progressive kidney function loss. Notably, hypertriglyceridemia (i.e., high circulating triglyceride concentrations) is common among individuals with OSA (Newman et al., 2001). These high concentrations may lead to ectopic lipid deposition, disruption of normal cellular functions, oxidative stress, systemic inflammation, and insulin resistance (Szendroedi & Roden, 2009). Multiple lines of evidence from in vitro and rodent models support the existence of high‐fat diet‐induced kidney injury, causing structural and functional impairments, accompanied by intracellular lipid deposition in podocytes and elevations in proinflammatory cytokines and proteinuria (Yu et al., 2022). There is, however, a lack of information linking acute elevations in circulating triglyceride concentrations with alterations in kidney function in humans. Conversely, considering the important role that insulin plays in the regulation of electrolyte balance by renal epithelial cells, it has been suggested that postprandial fluctuations in renal physiology may partly explain the relationship between some comorbidities (Blass et al., 2019). Specifically, Blass et al. (2019) demonstrated that insulin signaling mediates sodium absorption independently of the renin‐angiotensin‐aldosterone system. Although speculative, this high oxygen‐consuming process could aggravate the medulla's oxygen supply, especially during intermittent hypoxemia given that renal oxygen consumption does not appear to be influenced by even mild hypoxemia, potentially contributing to kidney hypoxia, and playing a role in the progression of kidney disease (Evans et al., 2011).

To date, the underlying mechanisms linking OSA with progressive loss of kidney function remain unclear in both acute and chronic fashions. Acute kidney injury (AKI) is defined as a transient (less than 7 days) reduction in kidney function and, while AKI biomarkers eventually return to baseline following AKI, the recovery of kidney function is often incomplete, and successive AKI can lead to the progressive deterioration of kidney function and ultimately CKD, end‐stage renal disease (ESRD), and death (Chawla et al., 2017). Reliable AKI biomarkers are sensitive to elevations in inflammation and oxidative stress and are therefore modulated within hours of kidney damage, such as during ischemia–reperfusion injury (Cruz et al., 2010). Most notably, there is emerging support for the use of neutrophil gelatinase‐associated lipocalin (NGAL), interleukin‐18 (IL‐18), liver‐type fatty acid‐binding protein (L‐FABP), and kidney injury molecule‐1 (KIM‐1) as AKI biomarkers. Consequently, we sought to retrospectively examine the effects of acute intermittent hypoxemia on circulating AKI biomarkers in individuals with and without OSA during the postprandial state. Considering that (1) elevated triglyceride levels and intermittent hypoxemia can lead to increased inflammation, oxidative stress, alterations in hemodynamics, and induction of the renin‐angiotensin system, (2) increasing insulin signaling leads to increased kidney function and oxygen consumption, and (3) chronic intermittent hypoxemia may predispose individuals with OSA to kidney disease, it was hypothesized that AKI biomarkers would increase following the consumption of a high‐fat meal in individuals with OSA and healthy individuals exposed to intermittent hypoxemia.

## 2 MATERIALS AND METHODS

### 2.1 Ethics statement

This study consisted of a single‐site, randomized crossover trial at the Behavioural and Metabolic Research Unit at the University of Ottawa. The Research Ethics Boards of the University of Ottawa and Montfort Hospital approved the protocol, agreeing with the Declaration of Helsinki, except for registration in a publicly accessible database (H‐06‐18‐837). All individuals provided written and informed consent before participating.

### 2.2 Participants

A total of 18 young men were recruited by convenience sampling from the University of Ottawa, and 7 individuals newly diagnosed with OSA were referred from the Montfort Hospital. Due to the confidential nature of polysomnography results, the sleep apnea parameters of the OSA group were not obtained and are therefore not reported. Previous data generated from some of these participants have been published elsewhere as part of a broader investigation into the effects of intermittent hypoxemia (Mahat et al., 2016; Morin et al., 2021). Participants were included if they habitually obtained 7–10 h of sleep per night and if they were aged 18–35 years for the healthy control group or aged 18–70 years for the OSA group. Participants were excluded if they had any history of respiratory illness, hypertension, cardiovascular disease, diabetes, current tobacco smoking status, or active use of lipid‐lowering medication. These eligibility criteria were chosen to control for confounding factors that may alter the postprandial response during intermittent hypoxemia.

### 2.3 Experimental design

Each participant completed a screening session and two experimental sessions. Participants were instructed to obtain a minimum of 7 h of sleep, and avoid exercise, caffeine, and alcohol within the 24 h preceding each session. Participants were also given a standardized dinner to consume 12 h before arriving at the laboratory. The experimental sessions were randomized, scheduled at the same time of day (ranging from 6:00 a.m. to 8:00 a.m.), and separated by at least 7 days.

#### 2.3.1 Screening session

Medical history, lifestyle habits, and anthropometric and metabolic characteristics were assessed during the screening session. Height was measured using a stadiometer (Perspective Enterprises, USA). Weight was measured using a beam scale (Tanita, USA). Body composition was measured using dual‐energy x‐ray absorptiometry (General Electric Lunar Prodigy, USA). Resting metabolic rate was measured using indirect calorimetry (VIASYS Healthcare Inc, USA) in a thermoneutral dark room for 30 min after a 12‐h overnight fast. Daily energy expenditure was estimated with the Harris and Benedict equation which multiplies resting metabolic rate by a physical activity factor of 1.375, corresponding to light exercise performed 1–3 days per week (Harris & Benedict, 1918). Each participant was exposed to intermittent hypoxemia using our experimental setup for up to 20 min and was asked to report any symptom listed in the Lake Louise Acute Mountain Sickness scoring system (Roach et al., 2018). All participants tolerated the hypoxic pre‐test, and no symptoms related to acute mountain sickness were reported.

#### 2.3.2 Experimental sessions

Upon arrival at the laboratory, an intravenous catheter was inserted by a certified phlebotomist via an antecubital venipuncture, and a baseline blood sample (0 min) was collected. Blood pressure was measured before each blood sample using an automatic sphygmomanometer (American Diagnostic Corporation, USA). Each experimental session consisted of consuming a high‐fat meal (59% fat, 33% of daily energy expenditure), containing a mixture of Ensure Plus (Abbott Laboratories, USA) and 35% whipping cream (Sealtest, Canada). Participants were instructed to remain awake in a semirecumbent position for 6 h. Water was allowed ad libitum.

Participants were equipped with a fingertip pulse oximeter (Masimo, USA), allowing heart rate and oxyhemoglobin saturation (S_p_O_2_) to be recorded every second. Following meal consumption, participants were randomly exposed to normoxia (20.93% oxygen, ~98% S_p_O_2_) or intermittent hypoxemia (~15 hypoxic cycles per hour: 100% nitrogen, ~85% S_p_O_2_) for 6 h. The hypoxic cycles were conducted using an oro‐nasal mask with a two‐way Hans Rudolph non‐rebreathing valve, with participants inhaling 100% medical grade nitrogen (Messer, Canada) until their S_p_O_2_ dropped to approximately 85%, at which point the inspiratory line was switched to ambient air (20.93% oxygen). S_p_O_2_ was allowed to return to baseline (~98%) before inducing the next hypoxic cycle. The frequency of hypoxic cycles of approximately 15 per hour represents a moderate score on the Apnea‐Hypopnea Index (AHI), a commonly used metric of OSA severity (Hudgel, 2016). The severity classifications are no sleep apnea (AHI <5), mild sleep apnea (AHI = 5–14), moderate sleep apnea (AHI = 15–30), and severe sleep apnea (AHI > 30).

### 2.4 Plasma analysis

Blood samples of approximately 20 mL were collected at 0, 30, 60, 90, 120, 180, 240, 300, and 360 min following meal consumption in tubes containing an ethylenediaminetetraacetic acid coating (BD Vacutainer, USA). Plasma was immediately separated from whole blood by centrifugation at 3000 rpm for 10 min at 4°C, then aliquoted and stored at −80°C until analysis. Colorimetric assays were used to determine plasma concentrations of glucose, triglycerides, and non‐esterified fatty acids (NEFA) (Wako Diagnostics, USA) and creatinine (Cayman Chemical, USA). Plasma concentrations of insulin, NGAL, IL‐18, KIM‐1, and L‐FABP were analyzed using enzyme‐linked immunosorbent assays (Bio‐Techne, Canada). For this secondary analysis, AKI biomarkers were only measured at 0, 180, and 360 min. Absorbance was read using a plate reader (Biotek, USA). Assays were performed per the manufacturer's instructions in duplicate. The detection ranges were: 0.0–38.9 mmol/L for glucose, 2.6–83.3 μIU/mL for insulin, 0.061–111.1 mmol/L for triglycerides, 0.0114–4.00 mmol/L for NEFA, and 0.5–5.0 mg/dL for creatinine, 78.1–5000 pg/mL for NGAL, 11.7–750 pg/mL for IL‐18, 15.6–1000 pg/mL for KIM‐1, and 125.0–2000 pg/mL for L‐FABP. The mean intra‐assay coefficient of variability was 4.5%.

### 2.5 Data analysis

To assess kidney function, glomerular filtration rate (eGFR) was estimated using the Chronic Kidney Disease Epidemiology Collaboration creatinine equation while considering race as “white and other” (Stevens et al., 2011). Additionally, we analyzed the absolute values and changes (∆) of all biomarkers as AKI is clinically defined by a reduction in kidney function, rather than by absolute concentrations. Using GPower (Version 3.1), an a priori power analysis was performed using a moderate effect size (Cohen, 1988), similar to a previous study using an effect size based on their NGAL data (*f* = 0.27) (Chapman et al., 2020). Using this effect size (*f* = 0.25) and a within‐participant correlation of 0.5, a total of 24 participants were required to achieve a power of 0.80 with an alpha set at 0.05 to identify a significant interaction (G × Power). Data are presented as mean (standard deviation or error) and, where appropriate, as individual points.

### 2.6 Statistical analysis

Participant characteristics were compared between groups (OSA × control) using unpaired, two‐tailed, *t*‐tests. All other variables were analyzed using a repeated measure analysis of variance with time and condition (normoxia × intermittent hypoxemia) as within‐participant parameters and group (OSA × control) as a between‐participant parameter while using the Greenhouse–Geisser correction. Identification of a significant interaction led to further analysis of simple main effects for a group or condition. Partial eta squared (*η*_p_^2^) was used to measure the proportion of variance attributed to the tested factor. Statistical analyses were conducted using jamovi (Version 2.2.5) with an alpha set at 0.05.

## 3 RESULTS

### 3.1 Participant characteristics

The physical characteristics of each experimental group are presented in Table 1. No between‐group differences were observed for weight and body mass index (*p* ≥ 0.074). However, age and fat mass (kg and %) were lower in the control group compared to the OSA group (*p* ≤ 0.023), while height and lean mass were higher in the control group compared to the OSA group (*p* ≤ 0.040).

**TABLE 1:** Physical characteristics per experimental group (OSA, *n* = 7; control, *n* = 18).

Characteristics	OSA (men, *n* = 6; women, *n* = 1)	Control (men, *n* = 18)	*p* value
Age (years)	54.4 (6.4)	22.7 (3.1)	<0.001
Height (m)	1.76 (0.1)	1.82 (0.1)	0.040
Weight (kg)	90.5 (22.6)	84.4 (10.7)	0.322
BMI (kg/m^2^)	29.4 (6.6)	25.4 (3.5)	0.074
Lean mass (kg)	57.7 (12.4)	66.4 (7.3)	0.040
Fat mass (kg)	30.2 (14.6)	16.4 (12.0)	0.023
Fat mass (%)	33.0 (8.7)	17.6 (7.6)	<0.001

*Note*: Data are presented as mean (standard deviation).

*Abbreviations*: BMI, body mass index; OSA, obstructive sleep apnea.

### 3.2 Physiological responses

Systolic and diastolic blood pressures, heart rate, and S_p_O_2_ are presented in Table 2. In both groups, no differences between conditions were observed in mean blood pressures (*p* ≥ 0.265); however, mean diastolic blood pressure was higher in the OSA group relative to the control group (*p* = 0.011, *η*_p_^2^ = 0.458). Across groups, mean heart rate was higher during intermittent hypoxemia relative to normoxia (*p* = 0.029, *η*_p_^2^ = 0.391), however further analysis revealed that this effect was only observed in the control group (P_tukey_ = 0.035). Compared to normoxia, every metric of S_p_O_2_ (i.e., mean, minimum, time spent under 90%, 85%, and 80%) was lower during intermittent hypoxemia (*p* ≤ 0.004, *η*_p_^2^ ≥ 0.839).

**TABLE 2:** Systolic and diastolic blood pressures, heart rate, and oxyhemoglobin saturation during 6 h of normoxia and intermittent hypoxemia in individuals with obstructive sleep apnea and healthy controls.

	OSA		Control		Group	Condition
	Normoxia	Hypoxia	Normoxia	Hypoxia	*p* value	*p* value
Mean SBP (mmHg)	136.0 (20.2)	134.5 (19.9)	124.6 (4.0)	128.4 (8.6)	0.285	0.505
Mean DBP (mmHg)	79.9 (10.2)	80.0 (8.8)	65.3 (6.1)	67.6 (5.4)	0.011	0.265
Heart rate (bpm)						
Mean	78.7 (11.0)	79.6 (7.0)	61.8 (14.1)	68.3 (14.9)	0.203	0.029
Maximum	118.3 (2.3)	98.7 (17.2)	97.8 (11.5)	102.5 (9.6)	0.308	0.156
S_p_O_2_ (%)						
Mean	94.1 (2.2)	91.9 (1.9)	96.8 (0.4)	93.8 (1.1)	0.070	0.002
Minimum	82.7 (6.4)	62.0 (7.2)	92.0 (2.2)	99.8 (0.5)	0.917	<0.001
Time S_p_O_2_ ≤ 90% (min)	33.2 (55.9)	76.3 (36.0)	0.1 (0.2)	53.2 (22.2)	0.270	0.004
Time S_p_O_2_ ≤ 85% (min)	0.5 (0.5)	26.3 (2.3)	0.0 (0.0)	26.0 (16.2)	0.935	0.003
Time S_p_O_2_ ≤ 80% (min)	0.1 (0.1)	13.5 (3.9)	0.0 (0.0)	12.7 (7.8)	0.855	0.003

*Note*: Data are presented as mean (standard deviation). Data for SBP, DBP, HR, and S_p_O_2_ include only complete time series (4 controls, 3 OSA) as technical issues were experienced with some recordings.

Abbreviations: DBP, diastolic blood pressure; OSA, obstructive sleep apnea; S_p_O_2,_ oxyhemoglobin saturation; SBP, systolic blood pressure.

### 3.3 Postprandial glycemia, insulinemia, and lipemia

**FIGURE 1:**
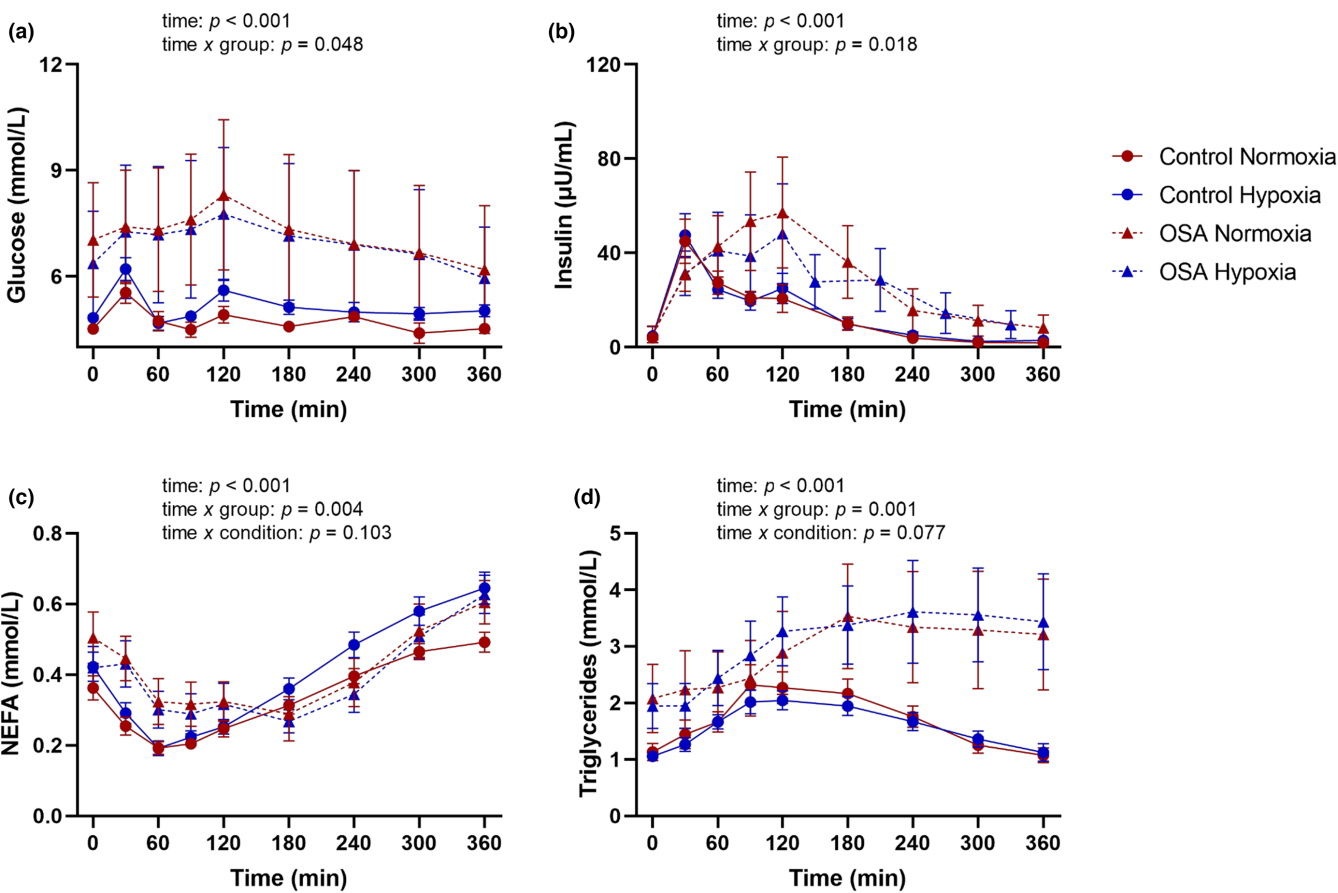
Plasma concentrations of glucose (a), insulin (b), non‐esterified fatty acids (NEFA) (c), and triglycerides (d) in individuals with obstructive sleep apnea (OSA) and healthy controls following the consumption of a high‐fat meal during 6 h of normoxia and intermittent hypoxemia. Data are presented as mean (standard error).

Baseline plasma concentrations of glucose, insulin, triglycerides, and NEFA are presented in Table 3. Baseline plasma concentrations of insulin and NEFA were similar across groups (*p* ≥ 0.330). Baseline plasma concentrations of glucose and triglycerides were higher in the OSA group compared to the control group (main effect of group: *p* ≤ 0.043, *η*_p_^2^ ≥ 0.166). Plasma concentrations of glucose, insulin, triglycerides, and NEFA across time are presented in Figure 1. Plasma concentrations of glucose and insulin evolved differently across time between groups (time × group interaction: *p* ≤ 0.048, *η*_p_^2^ ≥ 0.100), with peak concentrations occurring later in the OSA group relative to the control group, and remaining elevated over time. Plasma NEFA concentrations also evolved differently across time between groups (group × time interaction: *p* = 0.004, *η*_p_^2^ = 0.169), with higher plasma NEFA concentrations observed in the OSA group relative to the control group. Additionally, plasma triglyceride concentrations evolved differently across time between groups (group × time interaction: *p* = 0.001, *η*_p_^2^ = 0.407), remaining elevated in the OSA group in both conditions.

**TABLE 3:** Baseline values of estimated glomerular filtration rate and plasma concentrations of acute kidney injury biomarkers, glucose, insulin, non‐esterified fatty acids, and triglycerides in individuals with obstructive sleep apnea and healthy controls before 6 h of normoxia and intermittent hypoxemia.

	OSA		Control		Group	Condition
	Normoxia	Hypoxia	Normoxia	Hypoxia	*p* value	*p* value
Glucose (mmol/L)	7.0 (4.3)	6.4 (4.8)	4.5 (0.6)	4.8 (0.6)	0.043	0.281
Insulin (μU/mL)	5.5 (9.3)	5.3 (9.1)	4.0 (5.9)	4.7 (5.7)	0.708	0.829
NEFA (mmol/L)	0.5 (0.2)	0.4 (0.2)	0.4 (0.2)	0.4 (0.2)	0.330	0.702
Triglycerides (mmol/L)	1.9 (1.1)	2.1 (1.6)	1.1 (0.4)	1.2 (0.7)	0.023	0.482
eGFR (mL/min/1.73 m^2^)	69 (5)	73 (11)	92 (12)	92 (10)	<0.001	0.615
Creatinine (mg/dL)	2.0 (0.2)	1.9 (0.6)	1.8 (0.5)	1.8 (0.5)	0.437	0.740
NGAL (pg/mL)	6334 (4260)	5043 (2976)	5457 (2157)	5296 (2205)	0.772	0.170
IL‐18 (pg/mL)	392 (293)	361 (234)	307 (314)	328 (328)	0.669	0.832
L‐FABP (pg/mL)	3149 (1849)	2721 (1386)	2201 (1795)	2170 (1767)	0.332	0.213
KIM‐1 (pg/mL)	151 (137)	173 (172)	723 (811)	701 (807)	0.282	0.991

*Note*: Data are presented as mean (standard deviation).

Abbreviations: eGFR, estimated glomerular filtration rate; IL‐18, interleukin‐18; KIM‐1, kidney injury molecule‐1; L‐FABP, Liver‐type fatty acid‐binding proteins; NEFA, non‐esterified fatty acids; NGAL, neutrophil gelatinase‐associated lipocalin; OSA, obstructive sleep apnea.

### 3.4 Plasma biomarkers of acute kidney injury

**FIGURE 2:**
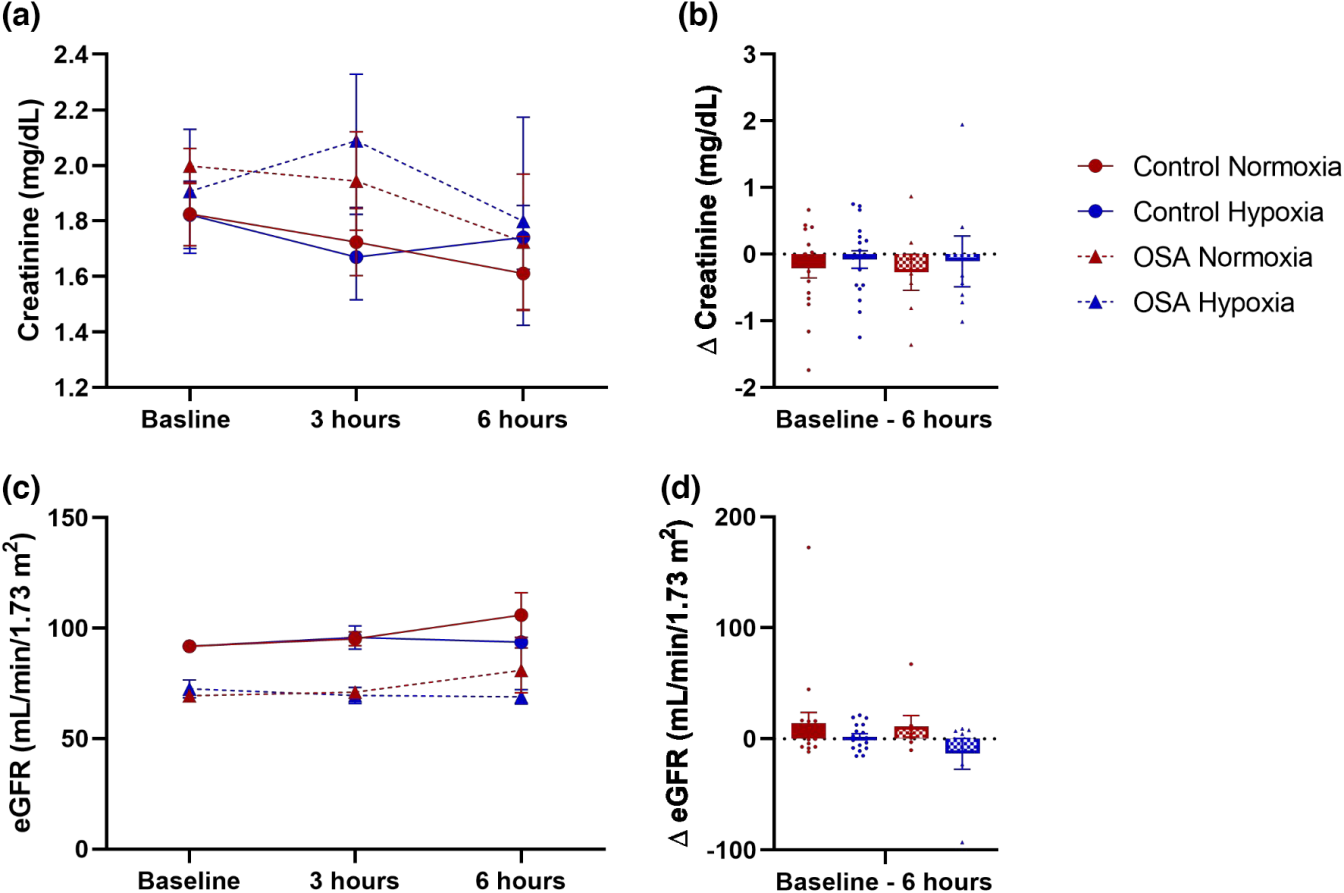
Plasma concentrations of creatinine as absolute values (a) and changes (∆) (b). Estimated glomerular filtration rate (eGFR) as absolute values (c) and changes (∆) (d) in individuals with obstructive sleep apnea (OSA) and healthy controls following the consumption of a high‐fat meal during 6 h of normoxia and intermittent hypoxemia. Data are presented as mean (standard error) and as individual points.

**FIGURE 3:**
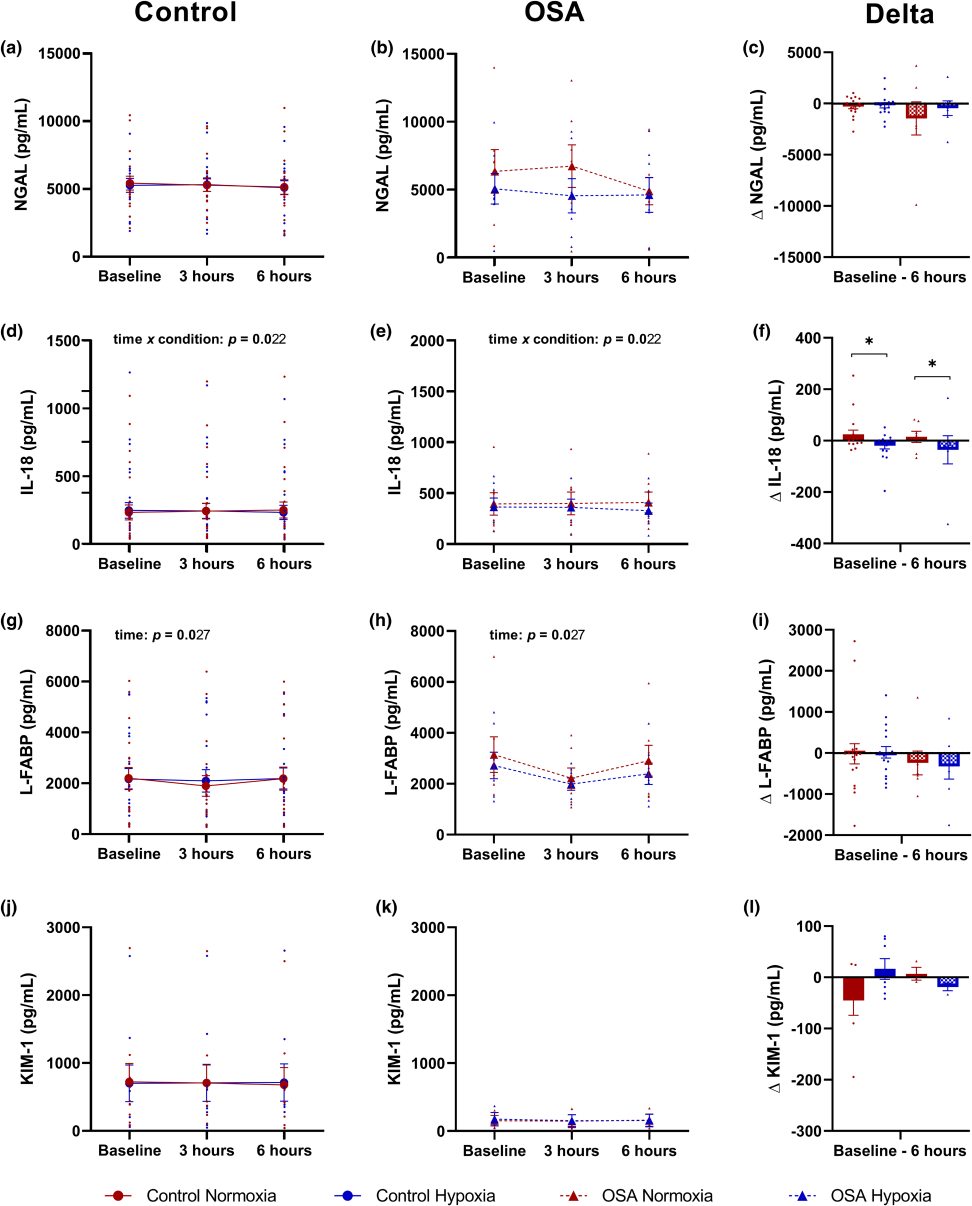
Absolute values and changes (∆) in plasma concentrations of acute kidney injury biomarkers in individuals with obstructive sleep apnea (OSA) and healthy controls following the consumption of a high‐fat meal during 6 h of normoxia and intermittent hypoxemia. Data are presented as mean (standard error) and as individual points. Statistical analyses represent pooled groups. Asterisk (*) indicates statistically different values than intermittent hypoxemia, *p* < 0.05. (a–c) Neutrophil gelatinase‐associated lipocalin (NGAL). (d–f) Interleukin‐18 (IL‐18). (g–i) Liver‐type fatty acid‐binding protein (L‐FABP). (j–l) Kidney injury molecule‐1 (KIM‐1).

Baseline values of eGFR and AKI biomarkers are presented in Table 3. Baseline eGFR was lower in the OSA group compared to the control group (main effect of group: *p* < 0.001, *η*_p_^2^ = 0.606). Conversely, baseline values of AKI biomarkers were similar across groups (*p* ≥ 0.282). Absolute values and changes in plasma concentrations of creatinine and eGFR are presented in Figure 2 and NGAL, IL‐18, KIM‐1, and L‐FABP are presented in Figure 3. Plasma concentrations of KIM‐1 for 15 participants were below the limit of detection (4 OSA; 11 control) at baseline and remained undetectable following the high‐fat meal with and without intermittent hypoxemia. These undetectable values were excluded from statistical analyses.

No significant time × condition × group or time × group interactions were detected for any AKI biomarker, plasma creatinine, and eGFR. Groups were thus pooled together for subsequent analyses. No effects of time or condition or time × condition interaction were observed for plasma concentrations of NGAL and KIM‐1. A significant time × condition interaction was observed for plasma IL‐18 concentrations (*p* = 0.022, *η*_p_^2^ = 0.175). When comparing the relative change between baseline and 6 h, plasma IL‐18 concentrations increased during normoxia compared to intermittent hypoxemia (*p* = 0.024, *η*_p_^2^ = 0.194). In both conditions, plasma L‐FABP concentrations were lower after 3 h and returned to baseline after 6 h (main effect of time: *p* = 0.027, *η*_p_^2^ = 0.142, *p*_tukey_ baseline vs. 3 h = 0.052, *p*_tukey_ baseline vs. 6 h = 0.794).

## 4 DISCUSSION

This retrospective analysis of an interventional study was aimed at evaluating the acute responses underlying the relationship between OSA and progressive kidney function loss. To our knowledge, this is the first investigation into the acute effects of a single high‐fat meal with or without a superimposed intermittent hypoxemia stress on kidney function. Contrary to our hypothesis, we demonstrated that plasma concentrations of AKI biomarkers do not increase within 6 h of exposure to intermittent hypoxemia in individuals with OSA or healthy young individuals following the consumption of a high‐fat meal. Conversely, we showed that plasma L‐FABP concentrations decreased after 3 h and returned to baseline values 6 h after meal consumption in both groups. Additionally, during normoxia, we showed that plasma IL‐18 concentrations increased in both groups following the consumption of a high‐fat meal.

Epidemiological studies show that the prevalence of CKD is high in individuals with OSA and, conversely, the prevalence of OSA is elevated in individuals with CKD, indicating a bidirectional relationship between OSA and CKD (Lin et al., 2020). The proposed mechanisms by which OSA leads to progressive loss of kidney function include nocturnal hypoxia, activation of the renin‐angiotensin system, increased sympathetic tone, endothelial dysfunction, inflammation, oxidative stress, and the presence of comorbidities such as hypertension, and diabetes mellitus (Voulgaris et al., 2019). While our experimental design allowed us to reduce S_p_O_2_ under 90% for 21% and 12% of total exposure time for the OSA group and the control group (respectively), we did not observe any reduction in eGFR or increase in plasma AKI biomarkers following 6 h of intermittent hypoxemia. In contrast, reductions in S_p_O_2_ below 90% for ≥12% of polysomnographic monitoring time have been associated with a ≥4 mL/min/1.73 m^2^ decline in eGFR per year (Ahmed et al., 2011). The discrepancy between our findings and those of Ahmed et al.'s (2011) may be explained by the acute nature of our intermittent hypoxemia protocol, as it does not reflect the chronicity of intermittent hypoxemia experienced by individuals living with OSA. More precisely, acute intermittent hypoxemia may not be sufficiently stressful to impair kidney function in the absence of other physiological disturbances such as fibrosis and microvasculature dysfunction. This is supported by the “chronic hypoxia hypothesis,” stating that the interaction of hypoxia with other factors influences the progression of CKD (Fine & Norman, 2008). Future research could investigate whether different OSA endotypes and phenotypes are more susceptible to renal injury following acute intermittent hypoxemia and whether this translates to a higher risk of developing kidney disease (Malhotra et al., 2020). While there were no observed changes in eGFR, we observed an increase in mean heart rate during intermittent hypoxemia relative to normoxia in healthy individuals, indicating an increase in sympathetic tone (Louis & Punjabi, 2009). The absence of change in heart rate in individuals with OSA concords with previous findings showing blunted cardiac responses in older adults following hypoxia (Lhuissier et al., 2012). We also did not observe any changes in blood pressure, suggesting that the renin‐angiotensin system or sympathetic tone were not sufficiently upregulated following the consumption of a high‐fat meal during normoxia or intermittent hypoxemia. Polysomnographic studies demonstrate that OSA leads to repetitive bursts in sympathetic nervous activity, which are accompanied by transient elevations in blood pressure (Somers et al., 1995). In the present study, the use of only nine measurements over 6 h may not have been sufficient to detect changes in blood pressure following intermittent hypoxic cycles.

Although the AKI biomarkers measured in this study are considered sensitive to ischemia–reperfusion injury and can be modulated within hours of kidney injury (Cruz et al., 2010), our findings suggest that moderate intermittent hypoxemia is not sufficient to acutely strain kidney function and increase plasma concentrations of NGAL, IL‐18, L‐FABP, and KIM‐1. While we did not observe any changes in plasma NGAL and KIM‐1 concentrations in either condition, we observed changes in plasma concentrations of L‐FABP during both conditions and changes in plasma concentrations of IL‐18 during normoxia. Specifically, plasma L‐FABP concentrations transiently decreased 3 h after meal consumption. L‐FABP is a cytoplasmic transporter for long‐chain fatty acids and promotes renoprotection by excreting lipid peroxidation products (Wang et al., 2005). In the absence of AKI, the observed reduction in plasma L‐FABP concentrations in the present study may be explained by the consumption of a high‐fat meal. The decrease in plasma concentrations of L‐FABP after 3 h of meal consumption mirrors the response pattern of plasma NEFA concentrations, which typically decrease shortly after a meal due to the anti‐lipolytic action of insulin (Stich & Berlan, 2004). While speculative, lower NEFA uptake during the first 3 h of the postprandial state may have decreased L‐FABP expression. Additionally, we observed an increase in plasma IL‐18 concentrations 6 h after meal consumption during normoxia, concurrent with previous findings showing an increase in serum IL‐18 concentrations 4 h after a high‐fat meal in individuals with and without type 2 diabetes (Esposito et al., 2003). Considering that a single high‐fat meal can transiently induce endothelial dysfunction (as assessed by flow‐mediated vasodilation) and increase circulating concentrations of inflammatory cytokines (Vogel et al., 1997), it remains unclear why the IL‐18 response in the present study was not observed during intermittent hypoxemia. To our knowledge, no study has investigated the mechanisms that regulate circulating IL‐18 concentrations during hypoxia in the postprandial state.

Altogether, the relationship between high‐fat diets, intermittent hypoxemia, and progressive loss of kidney function requires further investigation. In humans, high‐fat diets are associated with systemic inflammation and oxidative stress, as well as hemodynamic alterations and activation of the renin‐angiotensin system that may cause kidney strain (Felizardo et al., 2014). High‐fat diet‐induced kidney injury is also supported by the high prevalence of ESRD in individuals living with obesity (Ejerblad et al., 2006). While we did not observe any changes in biomarkers indicative of AKI, we found that postprandial triglyceride levels in individuals with OSA were substantially elevated compared to individuals without OSA, regardless of exposure to intermittent hypoxemia. It therefore remains plausible that recurrent exposure to high‐fat diet content and intermittent hypoxemia may play a more pronounced role in kidney disease, as incomplete recovery from recurrent episodes of kidney strain can lead to the progressive development of CKD (Chawla et al., 2017). Concurrently, recent reports indicate that sustained exposure to hypoxia in high altitudes can promote the progression of CKD to ESRD, although the relationship between high altitude and AKI has yet to be explored in depth, and it remains unclear if dietary factors can modulate this relationship (Wang et al., 2022). To date, there is no consensus on the best dietary recommendations for managing OSA and disease risk. Calorie‐restricted diets with low‐fat content have shown promising results in inducing weight loss and improving OSA severity (Araghi et al., 2013). However, there is also growing support for using low‐carbohydrate diets or the Mediterranean diet to improve overall metabolic health in individuals with OSA (Dobrosielski et al., 2017). Behavioural interventions such as regular exercise and healthy sleep hygiene should also be considered to improve postprandial triglyceride levels (Lopez‐Miranda et al., 2007). In summary, this study advances our understanding of physiological stressors (i.e., intermittent hypoxemia, postprandial state) that may acutely impact kidney function in humans. However, our findings are limited to the moderate intensity of intermittent hypoxemia employed and the confounding effect of a high‐fat meal. This study is also limited to assessing AKI biomarkers in plasma. Future studies should consider analyzing urine samples collected during intermittent hypoxemia and additional post‐exposure measurements, as AKI biomarkers can continue to increase over 2–3 days following initial kidney insult (Stanski et al., 2019). Finally, AKI biomarkers were only assessed in male individuals (except for one female individual with OSA) as the primary investigations controlled for biological sex‐related differences in postprandial responses in healthy young adults, yet accepted referrals for patients with a recent OSA diagnosis irrespective of gender or biological sex.

## 5 CONCLUSION

In conclusion, this exploratory study demonstrated that eGFR and plasma concentrations of NGAL, L‐FABP, and KIM‐1 did not increase following the consumption of a high‐fat meal in individuals with and without OSA during normoxia or intermittent hypoxemia. In contrast, plasma IL‐18 concentrations increased during normoxia. These findings suggest that acute intermittent hypoxemia does not cause sufficient kidney strain to acutely decrease kidney function in the postprandial state. While our study failed to demonstrate a clear relationship between intermittent hypoxemia and AKI biomarkers, it remains important to investigate how OSA modulates kidney function. Given the high prevalence of CKD in individuals with OSA and vice versa, further controlled studies are needed to elucidate the acute and chronic mechanisms underlying this relationship to ultimately develop better screening strategies and treatments for CKD.

## AUTHOR CONTRIBUTIONS

**Nicholas Goulet:** Methodology, Formal Analysis, Investigation, Writing—Original Draft, Visualization. **Emily J. Tetzlaff:** Investigation, Writing—Review & Editing. **Renée Morin:** Methodology, Investigation, Writing—Review & Editing. **Jean‐François Mauger:** Methodology, Investigation, Writing—Review & Editing. **Ruwan Amaratunga:** Conceptualization, Writing—Review & Editing, Funding Acquisition. **Glen P. Kenny:** Writing—Review & Editing, Supervision. **Pascal Imbeault:** Conceptualization, Writing—Review & Editing, Supervision, Funding Acquisition.

## FUNDING INFORMATION

This study was funded by grants from the Natural Sciences and Engineering Research Council of Canada (RGPIN‐2019‐04438) (funds held by Pascal Imbeault) and the Association Médicale Universitaire de l'Hôpital Montfort (2019‐A03 Sleep Apnea) (funds held by Ruwan Amaratunga & Pascal Imbeault). Pascal Imbeault is a recipient of a research chair from Institut du Savoir Montfort (2017‐022‐Chair‐PIMB). Nicholas Goulet is a recipient of a master's scholarship from the Institut du Savoir Montfort. Emily J. Tetzlaff was supported by a Natural Sciences and Engineering Research Council of Canada Graduate Scholarship (PGSD3‐545893‐2020) and a Mitacs Accelerate Fellowship.

## CONFLICT OF INTEREST STATEMENT

No conflicts of interest, financial or otherwise, are declared by the authors.

## Data Availability

The datasets used and/or analyzed during the current study are available from the corresponding author on reasonable request.
